# Metformin therapy in a hyperandrogenic anovulatory mutant murine model with polycystic ovarian syndrome characteristics improves oocyte maturity during superovulation

**DOI:** 10.1186/1757-2215-4-8

**Published:** 2011-05-23

**Authors:** Mary E Sabatini, Lankai Guo, Maureen P Lynch, Joseph O Doyle, HoJoon Lee, Bo R Rueda, Aaron K Styer

**Affiliations:** 1 Vincent Center for Reproductive Biology, Vincent Department of Obstetrics and Gynecology, Massachusetts General Hospital/Harvard Medical School, Boston, MA, USA

**Keywords:** polycystic ovarian syndrome, metformin, hyperinsulinemia, oocyte, superovulation

## Abstract

**Background:**

Metformin, an oral biguanide traditionally used for the treatment of type 2 diabetes, is widely used for the management of polycystic ovary syndrome (PCOS)-related anovulation. Because of the significant prevalence of insulin resistance and glucose intolerance in PCOS patients, and their putative role in ovulatory dysfunction, the use of metformin was touted as a means to improve ovulatory function and reproductive outcomes in PCOS patients. To date, there has been inconsistent evidence to demonstrate a favorable effect of metformin on oocyte quality and competence in women with PCOS. Given the heterogeneous nature of this disorder, we hypothesized that metformin may be beneficial in mice with aberrant metabolic characteristics similar to a significant number of PCOS patients. The aim of this study was to gain insight into the *in vitro *and *in vivo *effects of metformin on oocyte development and ovulatory function.

**Methods:**

We utilized metformin treatment in the transgenic *ob/ob *and *db/db *mutant murine models which demonstrate metabolic and reproductive characteristics similar to women with PCOS. Results: Metformin did not improve *in vitro *oocyte maturation nor did it have an appreciable effect on *in vitro *granulosa cell luteinization *(*progesterone production) in any genotype studied. Although both mutant strains have evidence of hyperandrogenemia, anovulation, and hyperinsulinemia, only *db/db *mice treated with metformin had a greater number of mature oocytes and total overall oocytes compared to control. There was no observed impact on body mass, or serum glucose and androgens in any genotype.

**Conclusions:**

Our data provide evidence to suggest that metformin may optimize ovulatory performance in mice with a specific reproductive and metabolic phenotype shared by women with PCOS. The only obvious difference between the mutant murine models is that the *db/db *mice have elevated leptin levels raising the questions of whether their response to metformin is related to elevated leptin levels and/or if a subset of PCOS women with hyperleptinemia may be responsive to metformin therapy. Further study is needed to better define a subset of women with PCOS that may be responsive to metformin.

## Background

Polycystic ovarian syndrome (PCOS) is a complex, multifactorial endocrinopathy which affects approximately 4 to 10% of reproductive-aged women. Because it is a highly heterogeneous syndrome with a variable clinical presentation, criteria for diagnosis have been debated. Many authorities utilize the guidelines of Rotterdam/ASRM-sponsored PCOS Consensus Workshop Group [1] and require the presence of at least two of the following: oligoovulation and/or anovulation, evidence of clinical or biochemical hyperandrogenism, and the presence of polycystic ovarian morphology during ultrasound examination.

PCOS is associated with several significant morbidities including infertility, obesity, insulin resistance, type 2 diabetes, dyslipidemia, and endometrial hyperplasia [2-6]. Proposed etiologies for PCOS include hypothalamic-pituitary dysynchrony, aberrant gonadotropin pulsatile secretion, granulosa/theca cell dysfunction, and various metabolic derangements including exaggerated ovarian androgen production, hyperinsulinemia, and insulin resistance [7-12]. Still, it is unclear whether the primary source of metabolic derangement is ovarian, hypothalamic/pituitary, or a combination of several systemic factors.

Several therapeutic options have been utilized to treat PCOS associated ovulatory dysfunction and infertility. These include weight loss, clomiphene citrate, exogenous gonadotropins, insulin sensitizers, and ovarian diathermy. Since its introduction as a treatment for type 2 diabetes in the United States in 1996, metformin also emerged as a common treatment for infertile women with PCOS [13-15]. Despite widespread and continued use, the efficacy of metformin as a treatment for PCOS remains unproven and controversial. Metformin has been shown by some investigators to result in weight loss, normalization of menstrual cycles, and an improvement of conception rates following therapies such as ovulation induction and controlled ovarian hyperstimulation prior to *in vitro *fertilization (IVF) [16-19]. In contrast, other studies have demonstrated that metformin does not offer any clinical benefit [20-22].

Metformin has been primarily characterized as an activator of AMP activated kinase (AMPK) [23]. AMPK serves as a sensor of energy status at the cellular level and is activated by an elevated AMP/ATP ratio. Activation of AMPK may induce catabolic processes which generate ATP and reduce anabolic processes which consume ATP. It can also serve as an energy sensor in several organs. For example, small decreases in glucose result in AMPK activation and decreased pancreatic insulin production with increase hypothalamic-driven feeding behavior [12,24-27]. Moreover, AMPK has evolved in higher organisms to be a highly complex regulator of cytokine function where leptin and adiponectin activate AMPK in muscle to increase glucose uptake and fatty acid oxidation [28,29]. The significance of metformin's role as an AMPK modulator is uncertain in reproductive processes such as oocyte maturation, ovulation, and luteinization.

To date, there is limited evidence demonstrating a consistent physiologic effect of metformin on oocyte development, ovulatory function, and fecundity in animal models. Previous data in the bovine model have demonstrated that metformin results in inhibition of maturation of denuded (DO) and non denuded oocytes. A similar effect was seen with a specific AMPK activator (AICAR), implying that metformin's inhibitory action may be mediated in part by AMPK activation in the oocyte [30]. Similarly, *in vitro *studies using porcine oocytes have shown that metformin prevents the maturation of the oocyte when it is part of the cumulus oophorus complex (COC). However, it did not prevent maturation of the porcine DO [31]. The AMPK activator, AICAR, has been shown to induce meiotic resumption in both mouse DO and COC *in vitro*, whereas this effect is blocked by Compound C, a specific AMPK inhibitor [32]. Metformin has also been shown to inhibit progesterone production *in vitro *through an AMPK mediated pathway in a number of cell types derived from several different species [33-35]. Notably, *in vitro *metformin concentrations of all aforementioned studies were supraphysiologic (0.1 - 2 mM). According to Lee and Kwon [36], serum concentrations in physiologic doses in humans are much lower, at approximately 8 - 10 μM.

Given the inconsistent results of published bovine and murine studies, and the controversy surrounding metformin's efficacy in PCOS-related ovulatory dysfunction and infertility, the goal of this study was to gain better insight into the effects of metformin on oocyte development and ovulation in mouse models which demonstrate metabolic and reproductive characteristics of women with PCOS. We utilized two different leptin mutant mouse strains. Both models, B6.Cg-m+/+ *Lep*^*ob*^/J or *ob/ob and the *B6.V-*Lep*^*db*^/J or *db/db*), exhibit obesity, hyperphagia, a diabetes-like syndrome of hyperglycemia, glucose intolerance, elevated plasma insulin, and subfertility [37,38]. The *ob/ob *strain does not produce endogenous leptin while the other strain, *db/db*, possess a nonfunctional leptin receptor and has elevated systemic leptin levels. We hypothesized that metformin therapy will have an effect on oocyte maturation and/or ovulatory function in *ob/ob *and *db/db *animals compared to wild type (WT) mouse strains.

## Methods

### Animal studies

#### Animals

Eight week-old female C57BL6 wild-type (WT), leptin deficient (B6.Cg-m+/+ *Lep*^*ob*^/J, *ob/ob*) and leptin receptor mutant (B6.V-*Lep*^*db*^/J, *db/db*) mice (Jackson Laboratory, Bar Harbor, ME) were housed in the animal facility at the Massachusetts General Hospital in accordance with the National Institutes of Health standards for the care and use of experimental animals. Rooms provided a controlled temperature range of (22-24°C) on a 14-hour light, 10-hour dark cycle. Mice were given food and water *ad libitum*. All animal procedures described were approved by the Subcommittee on Research Animal Care at Massachusetts General Hospital.

#### In vitro cultures

Thirty six to forty hours following injection of 10 IU pregnant mare serum gonadotropin (PMSG) (Sigma Aldrich, ST. Louis, MO, # G4877), animals were euthanized with intraperitoneal injection of Avertin 0.5 mg/ml followed by cervical dislocation, and the ovaries of each respective genotype were placed in DMEM supplemented with 10% fetal bovine serum (FBS). Follicles were punctured using a 28 gauge needle. For oocyte experiments, germinal vesicle (GV) oocytes were manually denuded with a glass pipette, pooled, and divided into DMEM with 10% FBS with or without insulin and/or varying concentrations of metformin. Metformin (Sigma Aldrich, St. Louis, MO, #D150959) for *in vitro *cultures was dissolved in Dulbecco's Modified Eagle's Medium (DMEM, Invitrogen 21063029, Carlsbad, CA) to 0.5 M, filter sterilized and diluted immediately into culture. Oocytes were incubated at 37°C with 5% O_2_. Maturity was assessed by light microscopy after 40 hours in culture. Oocytes were classified into the following groups: germinal vesicle oocytes (GV), germinal vesicle breakdown oocytes (GVBD), oocytes that have completed meiosis I (M1) (presence of first polar body) and fragmented (atretic) oocytes. Each experiment utilized 5 mice of each genotype (WT, *ob/ob, db/db*) with 15 oocytes in each *in vitro *metformin concentration group per replicate. Each experiment was performed in quadruplicate.

For granulosa cell experiments, ovaries were placed in phosphate buffered saline (PBS), and follicles were punctured as above. After manually removing residual ovarian tissue, the follicular contents were spun at 200 × g for 5 minutes at 4°C. The supernatant was removed and the pellets were resuspended in 1 mL of Weymouth's Solution (Invitrogen, Carslbad, CA, #11220035) supplemented with 10% FBS, Insulin-Transferrin-Selenium-A Supplement (Invitrogen, Carslbad, CA #51300-044, diluted 1:100), Penicillin-Streptomycin-Glutamine (Invitrogen, Carslbad, CA #10378, diluted 1:100) and sodium pyruvate (Invitrogen, Carslbad, CA #11360-070 diluted 1:100). Ten microliters was mixed with 10 μL of trypan blue and viable granulosa cells were counted with a hemocytometer. Cells were then diluted to a concentration of 5 × 10^4^/mL, and 1 mL was placed in a well of a 12 well plate. Culture medium was changed the following day (day 1 in culture) with the same medium except containing 1% FBS. Medium was changed every other day thereafter and frozen and stored as below.

#### Progesterone assays

Medium was removed from granulosa cell cultures on the day indicated and frozen at -20°C. Medium was then thawed and prepared per manufacturer's protocol (DRG EIA 1561, DRG International, Mountainside, NJ). Samples that contained greater than 40 ng/mL of progesterone underwent serial dilution so that readings fell within the standard curve of the assay (0.3 - 40 ng/mL) using a calibrated reader at 450 nm. Granulosa cells were pooled and subjected to treatments. Within each experiment, each sample was run in duplicate per manufacturer's recommendation. Each experiment was performed in triplicate.

#### Ovulation induction experiments

Six week old female mice were provided water alone or water which contained metformin at a concentration of 0.1 mg/ml for 7 weeks (treatment group). Because the murine estrous cycle is approximately 4.5 days, seven weeks is equivalent to approximately 12 estrous cycles. Metformin was added to daily water supply at a concentration of 0.1 mg/mL. Based upon the average water consumption of 6 mL of water per day of the C57BL6 mouse [39], this would amount to each mouse in the treatment group receiving a dose of metformin which approximates a standard adult human dose of 2,000 mg per day (28 mg/kg/day). Animals, which underwent superovulation, were injected with PMSG 10 IU IP followed 48 hours later by human chorionic gonadotropin (hCG) (Sigma Aldrich, St. Louis, MO, #CG10) 10 IU IP. Sixteen to eighteen hours after hCG treatment, serum glucose concentration was analyzed using a One Touch Ultra glucometer (LifeScan, Johnson and Johnson Subsidiary, 1000 Gibraltar Drive, Milpitas, CA). Subsequently, animals were weighed and euthanized with intraperitoneal injection of Avertin 0.5 mg/ml followed by cervical dislocation. Blood and oviducts were collected. Oocytes were removed from oviducts, counted and assessed for maturity and classified into the previously mentioned groups. Serum total testosterone was tested by radioimmunoassay (RIA) using the DPC Coat-A-Count RIA kit (Diagnostics Products Corporation, Los Angeles, CA). Experiments were performed in triplicate.

#### Ovarian follicular counts

Six-week old female mice were given metformin orally as above. At the end of the seven week period, animals were euthanized. Ovaries were dissected and were immediately fixed overnight in Deitricks fixative (0.34 N glacial acetic acid, 10% formalin, 28% ethanol) for histological assessment and processed for paraffin embedding. Serial sections (5 micrometers) were cut and dried for 24 hours. Sections were deparaffinized, rehydrated, and stained with hematoxylin for 10 minutes. Slides were counterstained with picric acid methyl blue for six minutes, dehydrated, coverslipped, and allowed to dry for 24 hours. Counts of primordial (single layer of flattened granulosa cells, preantral (single layer cuboidal granulosa cells), preantral (2-4 granulosa cell layers) and antral (> 4 granulosa cell layers with distinct antrum visible) follicles with visible nucleoli were performed on every fifth section in a blinded fashion according to previously described histomorphometric techniques [40,41]. Follicles were counted in 3 independent mice per genotype.

### Statistical analysis

Data were expressed as mean ± SEM of respective groups (experiments performed in triplicate or more as indicated). Data were analyzed using *t *test or two-way ANOVA with *post hoc *Tukey test. *P *< 0.01 designated as a statistically significant difference for ANOVA and *P *< 0.05 for comparison not using ANOVA.

## Results

### In vitro metformin treatment of mouse oocytes

**Figure 1:**
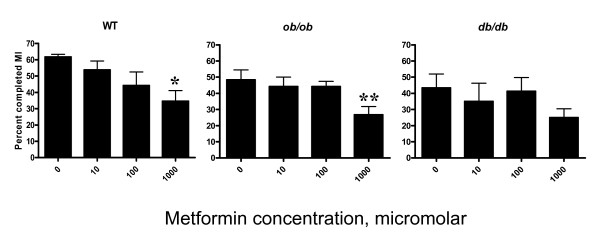
**Effect of metformin on *in vitro *oocyte maturation**. The vertical axis represents the percent of oocytes which completed meiosis I (MI) in culture. The horizontal axis shows *in vitro *metformin concentration (micromolar [μM]). Each experiment utilized 5 mice for each genotype (WT, *ob/ob, db/db*) with 15 oocytes in each *in vitro *metformin concentration group per replicate. Each experiment was performed in quadruplicate. Error bars are SEM. Oocytes treated with metformin concentration of 1000 μM demonstrated a reduction in percent oocytes which completed MI compared to control (0 μM) in WT and *ob/ob*. Asterisks indicate statistical significance of p < 0.05 for WT and *ob/ob *genotypes following comparison (* and ** *p *= 0.01, t test).

There was a significant difference in the percent of oocytes completing meiosis 1 (M1) in the 1000 μM treatment group relative to the control group (0 μM) in WT (p = 0.01), and *ob/ob *(p = 0.01) mice (Figure 1). The percent oocytes which completed M1 was 0.58 fold fewer in WT and 0.50 fold fewer in *ob/ob*. There was no difference in percent oocytes completing M1 *in vitro *in the *db/db *group.

### In vitro metformin exposure of mouse granulosa cell cultures

**Figure 2:**
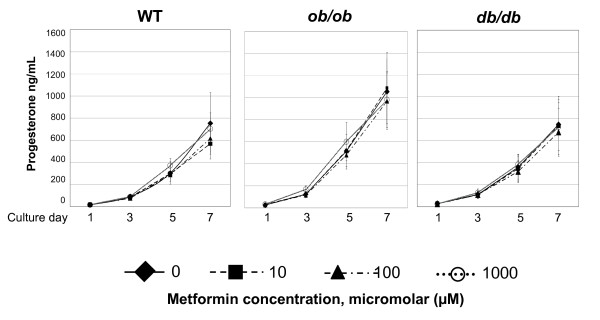
***In vitro *granulosa cell culture progesterone levels following exposure to metformin during seven days of metformin treatment**. Experiments were performed with 5 mice per genotype. Granulosa cells were pooled, divided into groups by metformin concentration and duration of culture, and media was collected for analysis. Experiments were performed in triplicate. Error bars are SEM. Compared to respective controls, no difference (P > 0.05, t test) was observed in progesterone levels of media in any metformin concentration during any time point in any genotype.

There was no significant difference in progesterone levels in the media of cultured granulosa cells treated with any concentration of metfomin at any time point assessed in any genotype relative to control (Figure 2).

### In vivo metformin treatment in WT, ob/ob, and db/db mice

**Figure 3:**
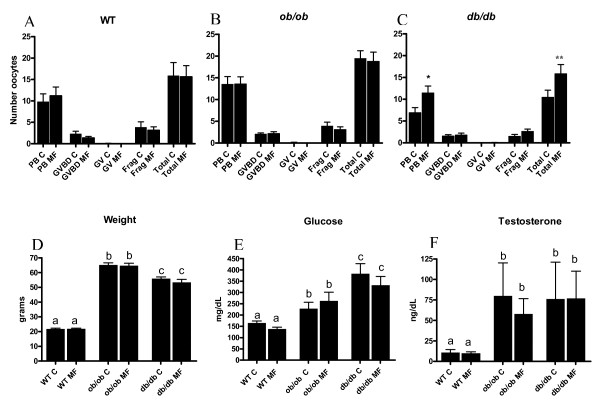
**Reproductive and metabolic effects of oral metformin pretreatment during superovulation**. For A-C, C = control, MF = metformin, PB = mature oocyte with polar body, GVBD = germinal vesicle break down oocyte, GV = immature germinal vesicle oocyte, frag = fragmented oocyte. Experiments were performed in triplicate. Error bars are SEM. A statistically significant increase in the quantity of ovulated mature oocytes (PB MF) and total number of oocytes ovulated (Total MF) was observed during superovulation in *db/db *mice compared to control (* denotes p = 0.018 and ** denotes p = 0.04, t test) (C). *ob/ob *and *db/db *mice demonstrated greater respective body mass (D) and testosterone levels (F) compared to WT mice. Metformin did not have an appreciable effect on any metabolic measure in any genotype relative to control (D, E, F). Different designated letters among genotypes in D, E, and F indicate statistical difference with *p *< 0.01 (ANOVA).

Following exposure with oral metformin for seven weeks (12 estrous cycles) and superovulation, significantly more mature oocytes and a greater total overall quantity of oocytes were recovered from *db/db *mice. Specifically, 1.77 fold more mature oocytes (p = 0.018) and 1.51 fold more total oocytes overall (p = 0.04) were obtained following superovulation of metformin exposed *db/db *mice relative to controls (no treatment) (Figure 3C). There was no difference in the quantity and proportion of mature oocytes obtained after superovulation of metformin exposed WT and *ob/ob *mice (Figure 3A, 3B). Animal weight (Figure 3D) and serum testosterone levels (Figure 3F) were unchanged during the metformin treatment course for any genotype. Blood glucose levels did not differ in response to metformin treatment in any genotype (Figure 3E). Both *ob/ob *and *db/db *mice demonstrated significantly greater baseline testosterone levels, serum glucose levels, and body mass (weight) than WT animals (p < 0.01).

### Gross ovarian anatomy

**Figure 4:**
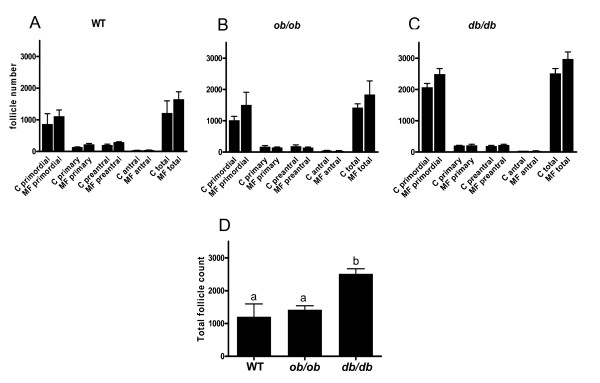
**Ovarian follicular counts (non atretic) following *in vivo *metformin exposure**. Horizontal axis indicates follicle stage. Follicle counts were performed in mice (N = 3) who underwent 7 weeks of oral metformin treatment or no treatment (control) (A, B, C). C = control, MF = metformin. All counts of this figure included 3 replicates with N = 1 mouse. Error bars represent SEM. Follicular counts (of any stage) did not change (relative to control group) following metformin treatment. Overall, *db/db *mice demonstrated a significantly greater total follicular endowment (sum of all non atretic follicle stages) than WT and *ob/ob *mice (D). Different designated letters among genotypes in D indicates statistical difference with p = 0.01 (ANOVA).

This analysis revealed that the control *db/db *mice demonstrated a two fold greater total non atretic follicular count relative to control WT animals (p = 0.01) (Figure 4D). Overall, animals treated with metformin did not demonstrate any change in the total follicle numbers or number in any specific follicular stage in any genotype studied compared to their respective controls.

## Discussion

Distinct features in women with PCOS, insulin resistance and compensatory hyperinsulinemia, lead to hyperandrogenemia due to increased ovarian androgen production and decreased production of sex hormone binding globulin [42,43]. Since hyperinsulinemia has been implicated as a significant cause of anovulation, many investigators hypothesized that a reduction of systemic insulin serum levels would result in an improvement of ovulatory function and overall fecundity in PCOS women. Initial studies investigating the use of metformin in PCOS demonstrated a beneficial role of metformin as an ovulation induction agent compared to placebo, clompihene citrate (CC), and CC and metformin combined [16]. However, two subsequent large, prospective, double blind studies did not demonstrate any benefit for metformin treatment in women with PCOS in terms of ovulation rate and pregnancy outcome [44,45]. Despite a long track record of metformin use in type 2 diabetes, it still remains unclear whether it provides a beneficial reproductive effect as an adjuvant therapy in women with PCOS. Furthermore, if there is a beneficial reproductive effect of metformin, it is unclear whether it acts locally at the level of the ovary, pituitary, hypothalamus, or on a more systemic level. In this study, we have demonstrated for the first time, that metformin confers significant *in vitro *and *in vivo *effects on oocyte maturation in mouse strains with metabolic and reproductive characteristics of PCOS. Specifically, we demonstrate a reduction in the completion of meiosis 1 by oocytes *in vitro *following metformin exposure in WT and *ob/ob *mice, and an increase in the yield of mature oocytes and total overall oocytes following continuous dietary metformin for 7 weeks prior to superovulation in a *db/db in vivo *model.

We hypothesized that treatment with the insulin sensitizer, metformin, would have an impact on oocyte maturation and/or ovulation in a PCOS-like mouse strain with a hyperinsulinemic and anovulatory phenotype. Previous studies examining the effects of metformin, have focused on specific compartments of the ovary, namely the oocyte and granulosa cells in WT animals (congenic mice and outbred strains of cows and pigs), with normal ovulatory function [30-32]. *In vitro *studies have demonstrated direct effects of metformin on the ovary, which involve inhibition of basal and insulin stimulated granulosa cell P450 aromatase via MEK/ERK (MAPK kinase) activation [46]. Similar to previously published studies detailing an inhibitory effect of metformin on *in vitro *oocyte maturation [30,31], the results of this study demonstrated that metformin reduced *in vitro *maturation of the mouse oocyte. Specifically, meformin exerted a significant reduction of maturation of oocytes derived from WT and *ob/ob *mice, but not in *db/db *mice. Notably, the *in vitro *concentration of metformin which demonstrated this finding was at the highest concentration, and may represent an extremely elevated *in vivo *serum level which surpasses the typical human metformin dose of 2000 mg daily dose (approximately 10 μM). These collective findings raise the possibility that this effect may be an artifact of toxicity of the high levels of metformin. Alternatively, these findings may be the result of *in vitro *conditions, which may not be directly applicable to *in vivo *conditions.

Based upon previous data [47] which demonstrated an antiapoptotic effect of metformin on luteinized granulosa cells in PCOS patients undergoing IVF, it may be expected that metformin treatment would result in elevated progesterone levels in conditioned media from cultured granulosa cells derived from both transgenic mouse models which share PCOS characteristics. However, there was no obvious effect of increasing doses of metformin on progesterone levels in conditioned media derived from granulosa cells in any genotype. Therefore, it can be inferred that there was no significant change in cell number. The differences in our results may be attributed to species to species variability in response to metformin or may reflect the complexity of steroidogenesis, which likely involves multiple pathways independent of those regulated by metformin.

*In vivo *studies examined the chronic effects of metformin pretreatment on oocyte development and ovulatory performance in WT, *ob/ob *and *db/db *mouse strains during superovulation. With the use of 0.1 mg/ml metformin in drinking water (approximate to human dose of 2000 mg per day), these experiments demonstrated that metformin significantly increased the number of mature oocytes ovulated by 1.77 fold (p = 0.018) and the total overall number of oocytes released by 1.51 fold (p = 0.04) in *db/db *mice during superovulation. Interestingly, this same result was not observed in the *ob/ob *mouse strain, which shares many phenotypic similarities (obesity, hyperglycemia, hyperinsulinemia, and infertility with anovulation). In contrast to the *ob/ob *mouse, which lack endogenous leptin production, the *db/db *mouse has elevated systemic leptin levels. An explanation of the results seen only in the *db/db *strain may be due to a possible effect of metformin on this animal's endogenously elevated leptin levels. Notably, there are preliminary data describing the reduction of leptin by metformin in women with PCOS [48]. However, the fact that the *db/db *mice lack a functional cognate receptor leptin receptor (long isoform) would imply that any change incurred by a decrease in leptin may be indicative of leptin eliciting a response through the less characterized short form of the OB receptor or via an unrecognized alternative receptor.

Given the known role of hyperinsulinemia and hyperandrogenemia in PCOS anovulation, it may also be initially inferred that the metformin treated *db/db *genotype displayed improved glucose control and weight loss compared to other mouse strains. However, there were no significant differences in weight, glucose, or testosterone levels in any metformin treated mouse strain compared to controls. This observation in the *db/db *mouse may signify a more pronounced, yet less detectable intrafollicular effect of hyperinsulinemia in this transgenic genotype. In line with prior observations of dysfunctional steroidogenesis and folliculogenesis in PCOS [49], correction of this metabolic derangement with the insulin sensitizer, metformin, may have established a more favorable intrafollicular insulin environment and may have optimized ovulatory performance, resulting in an improvement in the production of mature oocytes during superovulation in the *db/db *strain. Several authors have recently published findings which support a possible direct impact of metformin on the ovary. Stimulation of lactate production and activation of AMPK in granulosa cells by this compound has been proposed as a mechanism of improving follicular and oocyte development [50]. Additionally, the findings of Palomba *et al. *demonstrate a significant effect of metformin on intrafollicular insulin growth factor 2, several insulin growth factor binding proteins, estradiol, and androgen levels in women with PCOS [51].

Although there is not a single ideal animal model for PCOS, several reproductive and metabolic features commonly observed in PCOS are present in the animal models utilized in the present study. As highlighted previously, there are other additional mouse and rat models which have been utilized to study PCOS [52]. Unfortunately, some primarily possess metabolic traits, others demonstrate only reproductive characteristics, while others possess some combination of both [37,38,52,53]. As is true with other models, the mouse strains used in this study do not perfectly simulate human PCOS. To this end, one model will not be completely representative of all human PCOS phenotypes. Investigation in many different models will be likely required to gain a more comprehensive understanding of the metabolic and reproductive aspects of this syndrome. Since the *ob/ob *and *db/db *mice share both reproductive and metabolic characteristics of women with PCOS, it was most appropriate to utilize these strains to investigate the potential reproductive effects of metformin in a hyperinsulienmic and anovulatory *in vivo *model. Although the exact mechanism of metformin has not been elucidated, it has been shown to be an activator of AMPK. The inhibitory effects of metformin at the level of the oocyte have been inferred from various mammalian studies using the AMPK activator (AICAR) and AMPK inhibitor Compound C [30-32]. Unfortunately, it is difficult to directly assess the discreet physiologic role of metformin AMPK activation in reproduction in this model. In future studies, it may be possible to assess the role of the metformin AMPK pathway in another model since a group of investigators have demonstrated that the kinase LKB1 mediates glucose homeostasis in liver and the therapeutic effects of metformin [54]. In order to definitively characterize the function of metformin via the AMPK pathway, the use of the LKB1 deficient mouse may provide additional insight into AMPK mediated local and systemic effects of metformin from a metabolic and reproductive standpoint.

Due to the wide variation of metabolic and reproductive characteristics in women with the polycystic ovarian syndrome, it has become a difficult task to identify if any PCOS phenotype may benefit from metformin. The unpredictable extent to which a specific end organ is affected by insulin resistance or hyperinsulemia (e.g. ovary of a woman with PCOS) is likely contributory to the inconsistent results of previous studies examining metformin use in PCOS [54]. Given the continued uncertainty regarding the clinical reproductive benefit of metformin use for PCOS associated infertility, a study such as this, can assist the field in determining whether this adjuvant therapy is of tangible benefit in clinical practice. In the hyperinsulinemic and hyperandrogenic anovulatory leptin *ob/ob *and *db/db *mutant mouse strains, no significant effect of metformin was observed at physiologic levels *in vitro *at the level of oocyte or granulosa cells to increase oocyte maturity or progesterone production respectively. As hypothesized, a beneficial *in vivo *effect was demonstrated in the *db/db *strain as seen by an improvement of the yield of mature oocytes during superovulation. When considering our findings, it may be reasonable to speculate that metformin may act to optimize oocyte development and production by the local and/or systemic reduction of hyperinsulinemia, androgen and leptin production, as well as by the reduction of inappropriately high intrafollicular estradiol levels (seen in PCOS patients) by attenuation of aromatase activity as highlighted previously [46,49]. In light of recent findings which suggest that metformin may act via an insulin dependent mechanism in the human ovary, this treatment may confer a significant effect on oocyte development and ovulatory performance in the *dbdb *mouse and a subset of similarly hyperleptinemic and hyperinsulinemic women with PCOS [55], Additionally, the larger follicular endowment of *db/db *mice, compared to other genotypes, may also contribute an unknown influence on oocyte maturation and development during superovulation.

## Conclusions

In summary, by using transgenic mouse models with characteristics of PCOS, we have demonstrated a significant *in vivo *reproductive effect of metformin use in a specific mouse strain. These findings may imply that a specific subset of women with the PCOS reproductive phenotype may potentially benefit from metformin, while the majority with this syndrome will not. Additionally, the observed *in vivo *effects of metformin in the hyperleptinemic *db/db *strain may infer that a subset with the PCOS reproductive phenotype characterized by hyperinsulinemia, anovulation, and hyperleptinemia may be more responsive to metformin than those without elevated leptin levels. With the use of a transgenic mouse strain such as *db/db*, our findings demonstrate a possible role of metformin to optimize ovulatory performance during superovulation in mice with a specific reproductive and metabolic phenotype. To this end, future studies utilizing the *db/db *mouse strain and other PCOS-like murine models will provide the foundation for future investigation to clearly determine the utility of metformin treatment in the human model of PCOS.

The authors declare that they have no competing interests.

## Authors' contributions

MES cared for all animals used in the study, performed a majority of all i*n vitro *and *in vivo *experiments, and participated in manuscript preparation. All statistical analysis was performed by MES and reviewed by AKS and BRR. LG conducted *in vitro *progesterone assays and ovarian follicular counting experiments. MPL contributed to the conception and formulation of the study design and and to critical analysis of results. JOD assisted with animal care and progesterone assays. HJL served as an additional participant during the assessment of ovarian maturity in the *in vitro *studies, oocyte counts, and assessments during the superovulation studies. BRR participated in the conception and design of the study, critical analysis of data, and manuscript preparation. AKS is responsible for the original conception of the study, coordination and supervision of experiments, critical analysis of data, and preparation of the manuscript. All authors read and approved the final manuscript.

## References

[B1] Revised 2003 consensus on diagnostic criteria and long-term health risks related to polycystic ovary syndrome (PCOS) Hum Reprod 2004 19 41 47 14688154 10.1093/humrep/deh098

[B2] Goodarzi MO Azziz R Diagnosis, epidemiology, and genetics of the polycystic ovary syndrome Best Pract Res Clin Endocrinol Metab 2006 20 193 205 10.1016/j.beem.2006.02.005 16772151

[B3] Park KH Kim JY Ahn CW Song YD Lim SK Lee HC Polycystic ovarian syndrome (PCOS) and insulin resistance Int J Gynaecol Obstet 2001 74 261 267 10.1016/S0020-7292(01)00442-8 11543750

[B4] Apridonidze T Essah PA Iuorno MJ Nestler JE Prevalence and characteristics of the metabolic syndrome in women with polycystic ovary syndrome J Clin Endocrinol Metab 2005 90 1929 1935 10.1210/jc.2004-1045 15623819

[B5] Talbott EO Zborowski JV Rager JR Boudreaux MY Edmundowicz DA Guzick DS Evidence for an association between metabolic cardiovascular syndrome and coronary and aortic calcification among women with polycystic ovary syndrome J Clin Endocrinol Metab 2004 89 5454 5461 10.1210/jc.2003-032237 15531497

[B6] Pillay OC Te Fong LF Crow JC Benjamin E Mould T Atiomo W Menon PA Leonard AJ Hardiman P The association between polycystic ovaries and endometrial cancer Hum Reprod 2006 21 924 929 16361289 10.1093/humrep/dei420

[B7] Voutilainen R Franks S Mason HD Martikainen H Expression of insulin-like growth factor (IGF), IGF-binding protein, and IGF receptor messenger ribonucleic acids in normal and polycystic ovaries J Clin Endocrinol Metab 1996 81 1003 1008 10.1210/jc.81.3.1003 8772565

[B8] Burger CW Korsen T van Kessel H van Dop PA Caron FJ Schoemaker J Pulsatile luteinizing hormone patterns in the follicular phase of the menstrual cycle, polycystic ovarian disease (PCOD) and non-PCOD secondary amenorrhea J Clin Endocrinol Metab 1985 61 1126 1132 10.1210/jcem-61-6-1126 3932449

[B9] Berga SL Yen SS Opioidergic regulation of LH pulsatility in women with polycystic ovary syndrome Clin Endocrinol (Oxf) 1989 30 177 184 10.1111/j.1365-2265.1989.tb03739.x 2532984

[B10] Ehrmann DA Polycystic ovary syndrome N Engl J Med 2005 352 1223 1236 10.1056/NEJMra041536 15788499

[B11] Chang RJ A practical approach to the diagnosis of polycystic ovary syndrome Am J Obstet Gynecol 2004 191 713 717 10.1016/j.ajog.2004.04.045 15467530

[B12] Dunaif A Graf M Mandeli J Laumas V Dobrjansky A Characterization of groups of hyperandrogenic women with acanthosis nigricans, impaired glucose tolerance, and/or hyperinsulinemia J Clin Endocrinol Metab 1987 65 499 507 10.1210/jcem-65-3-499 3305551

[B13] Nestler JE Role of hyperinsulinemia in the pathogenesis of the polycystic ovary syndrome, and its clinical implications Semin Reprod Endocrinol 1997 15 111 122 10.1055/s-2007-1016294 9165656

[B14] Barbieri RL Gargiulo AR Metformin for the treatment of the polycystic ovary syndrome Minerva Ginecol 2004 56 63 79 14973411

[B15] Palomba S Oppedisano R Tolino A Orio F Zullo F Outlook: metformin use in infertile patients with polycystic ovary syndrome: an evidence-based overview Reprod Biomed Online 2008 16 327 335 10.1016/S1472-6483(10)60592-5 18339252

[B16] Lord JM Flight IH Norman RJ Metformin in polycystic ovary syndrome: systematic review and meta-analysis BMJ 2003 327 951 953 10.1136/bmj.327.7421.951 14576245 PMC259161

[B17] Velazquez EM Mendoza S Hamer T Sosa F Glueck CJ Metformin therapy in polycystic ovary syndrome reduces hyperinsulinemia, insulin resistance, hyperandrogenemia, and systolic blood pressure, while facilitating normal menses and pregnancy Metabolism 1994 43 647 654 10.1016/0026-0495(94)90209-7 8177055

[B18] Palomba S Orio F Jr Falbo A Russo T Tolino A Zullo F Clomiphene citrate versus metformin as first-line approach for the treatment of anovulation in infertile patients with polycystic ovary syndrome J Clin Endocrinol Metab 2007 92 3498 3503 10.1210/jc.2007-1009 17595241

[B19] Brewer C Acharya S Thake F Tang T Balen A Effect of metformin taken in the 'fresh' in vitro fertilization/intracytoplasmic sperm injection cycle upon subsequent frozen embryo replacement in women with polycystic ovary syndrome Hum Fertil (Camb) 2010 13 134 142 10.3109/14647273.2010.504805 20849198

[B20] Creanga AA Bradley HM McCormick C Witkop CT Use of metformin in polycystic ovary syndrome: a meta-analysis Obstet Gynecol 2008 111 959 968 10.1097/AOG.0b013e31816a4ed4 18378757

[B21] Legro RS Barnhart HX Schlaff WD Carr BR Diamond MP Carson SA Steinkampf MP Coutifaris C McGovern PG Cataldo NA Clomiphene, metformin, or both for infertility in the polycystic ovary syndrome N Engl J Med 2007 356 551 566 10.1056/NEJMoa063971 17287476

[B22] Tso LO Costello MF Albuquerque LE Andriolo RB Freitas V Metformin treatment before and during IVF or ICSI in women with polycystic ovary syndrome Cochrane Database Syst Rev 2009 CD006105 10.1002/14651858.CD006105.pub2 19370625

[B23] Zhou G Myers R Li Y Chen Y Shen X Fenyk-Melody J Wu M Ventre J Doebber T Fujii N Role of AMP-activated protein kinase in mechanism of metformin action J Clin Invest 2001 108 1167 1174 11602624 10.1172/JCI13505 PMC209533

[B24] Salt IP Johnson G Ashcroft SJ Hardie DG AMP-activated protein kinase is activated by low glucose in cell lines derived from pancreatic beta cells, and may regulate insulin release Biochem J 1998 335 Pt 3 533 539 9794792 10.1042/bj3350533 PMC1219813

[B25] da Silva Xavier G Leclerc I Salt IP Doiron B Hardie DG Kahn A Rutter GA Role of AMP-activated protein kinase in the regulation by glucose of islet beta cell gene expression Proc Natl Acad Sci USA 2000 97 4023 4028 10.1073/pnas.97.8.4023 10760274 PMC18135

[B26] Minokoshi Y Alquier T Furukawa N Kim YB Lee A Xue B Mu J Foufelle F Ferre P Birnbaum MJ AMP-kinase regulates food intake by responding to hormonal and nutrient signals in the hypothalamus Nature 2004 428 569 574 10.1038/nature02440 15058305

[B27] Andersson U Filipsson K Abbott CR Woods A Smith K Bloom SR Carling D Small CJ AMP-activated protein kinase plays a role in the control of food intake J Biol Chem 2004 279 12005 12008 14742438 10.1074/jbc.C300557200

[B28] Minokoshi Y Kim YB Peroni OD Fryer LG Muller C Carling D Kahn BB Leptin stimulates fatty-acid oxidation by activating AMP-activated protein kinase Nature 2002 415 339 343 10.1038/415339a 11797013

[B29] Yamauchi T Kamon J Minokoshi Y Ito Y Waki H Uchida S Yamashita S Noda M Kita S Ueki K Adiponectin stimulates glucose utilization and fatty-acid oxidation by activating AMP-activated protein kinase Nat Med 2002 8 1288 1295 10.1038/nm788 12368907

[B30] Bilodeau-Goeseels S Sasseville M Guillemette C Richard FJ Effects of adenosine monophosphate-activated kinase activators on bovine oocyte nuclear maturation in vitro Mol Reprod Dev 2007 74 1021 1034 10.1002/mrd.20574 17290417

[B31] Mayes MA Laforest MF Guillemette C Gilchrist RB Richard FJ Adenosine 5'-monophosphate kinase-activated protein kinase (PRKA) activators delay meiotic resumption in porcine oocytes Biol Reprod 2007 76 589 597 10.1095/biolreprod.106.057828 17167165

[B32] Chen J Hudson E Chi MM Chang AS Moley KH Hardie DG Downs SM AMPK regulation of mouse oocyte meiotic resumption in vitro Dev Biol 2006 291 227 238 10.1016/j.ydbio.2005.11.039 16443210

[B33] Tosca L Solnais P Ferre P Foufelle F Dupont J Metformin-induced stimulation of adenosine 5' monophosphate-activated protein kinase (PRKA) impairs progesterone secretion in rat granulosa cells Biol Reprod 2006 75 342 351 10.1095/biolreprod.106.050831 16760380

[B34] Tosca L Chabrolle C Uzbekova S Dupont J Effects of metformin on bovine granulosa cells steroidogenesis: possible involvement of adenosine 5' monophosphate-activated protein kinase (AMPK) Biol Reprod 2007 76 368 378 10.1095/biolreprod.106.055749 17123942

[B35] Tosca L Rame C Chabrolle C Tesseraud S Dupont J Metformin decreases IGF1-induced cell proliferation and protein synthesis through AMP-activated protein kinase in cultured bovine granulosa cells Reproduction 2010 139 409 418 10.1530/REP-09-0351 19906888

[B36] Lee SH Kwon KI Pharmacokinetic-pharmacodynamic modeling for the relationship between glucose-lowering effect and plasma concentration of metformin in volunteers Arch Pharm Res 2004 27 806 810 10.1007/BF02980152 15357011

[B37] Coleman DL Hummel KP The influence of genetic background on the expression of the obese (Ob) gene in the mouse Diabetologia 1973 9 287 293 10.1007/BF01221856 4588246

[B38] Coleman DL Obese and diabetes: two mutant genes causing diabetes-obesity syndromes in mice Diabetologia 1978 14 141 148 10.1007/BF00429772 350680

[B39] Bachmanov AA Reed DR Beauchamp GK Tordoff MG Food intake, water intake, and drinking spout side preference of 28 mouse strains Behav Genet 2002 32 435 443 10.1023/A:1020884312053 12467341 PMC1397713

[B40] Tilly JL Ovarian follicle counts--not as simple as 1, 2, 3 Reprod Biol Endocrinol 2003 1 11 10.1186/1477-7827-1-11 12646064 PMC151785

[B41] Legro RS Barnhart HX Schlaff WD Carr BR Diamond MP Carson SA Steinkampf MP Coutifaris C McGovern PG Cataldo NA Ovulatory response to treatment of polycystic ovary syndrome is associated with a polymorphism in the STK11 gene J Clin Endocrinol Metab 2008 93 792 800 18000088 10.1210/jc.2007-1736 PMC2266955

[B42] Dunaif A Insulin resistance and the polycystic ovary syndrome: mechanism and implications for pathogenesis Endocr Rev 1997 18 774 800 10.1210/er.18.6.774 9408743

[B43] Yu Ng EH Ho PC Polycystic ovary syndrome in asian women Semin Reprod Med 2008 26 14 21 10.1055/s-2007-992920 18181078

[B44] Moll E Bossuyt PM Korevaar JC Lambalk CB van der Veen F Effect of clomifene citrate plus metformin and clomifene citrate plus placebo on induction of ovulation in women with newly diagnosed polycystic ovary syndrome: randomised double blind clinical trial BMJ 2006 332 1485 10.1136/bmj.38867.631551.55 16769748 PMC1482338

[B45] Allahbadia GN Merchant R Polycystic ovary syndrome in the Indian Subcontinent Semin Reprod Med 2008 26 22 34 10.1055/s-2007-992921 18181079

[B46] Rice S Pellatt L Ramanathan K Whitehead SA Mason HD Metformin inhibits aromatase via an extracellular signal-regulated kinase-mediated pathway Endocrinology 2009 150 4794 4801 10.1210/en.2009-0540 19574398 PMC2749730

[B47] Onalan G Selam B Baran Y Cincik M Onalan R Gunduz U Ural AU Pabuccu R Serum and follicular fluid levels of soluble Fas, soluble Fas ligand and apoptosis of luteinized granulosa cells in PCOS patients undergoing IVF Hum Reprod 2005 20 2391 2395 10.1093/humrep/dei068 15932917

[B48] Marciniak A Nawrocka-Rutkowska J Brodowska A Sienkiewicz R Szydlowska I Starczewski A Leptin concentrations in patients with polycystic ovary syndrome before and after met-formin treatment depending on insulin resistance, body mass index and androgen con-centrations--introductory report Folia Histochem Cytobiol 2009 47 323 328 10.2478/v10042-009-0032-0 19995720

[B49] Franks S Gilling-Smith C Watson H Willis D Insulin action in the normal and polycystic ovary Endocrinol Metab Clin North Am 1999 28 361 378 10.1016/S0889-8529(05)70074-8 10352923

[B50] Richardson MC Ingamells S Simonis CD Cameron IT Sreekumar R Vijendren A Sellahewa L Coakley S Byrne CD Stimulation of lactate production in human granulosa cells by metformin and potential involvement of adenosine 5' monophosphate-activated protein kinase J Clin Endocrinol Metab 2009 94 670 677 10.1210/jc.2008-2025 19001513

[B51] Palomba S Falbo A Russo T Orio F Tolino A Zullo F Systemic and local effects of metformin administration in patients with polycystic ovary syndrome (PCOS): relationship to the ovulatory response Hum Reprod 2010 25 1005 1013 10.1093/humrep/dep466 20106839

[B52] Shi D Dyck MK Uwiera RR Russell JC Proctor SD Vine DF A unique rodent model of cardiometabolic risk associated with the metabolic syndrome and polycystic ovary syndrome Endocrinology 2009 150 4425 4436 10.1210/en.2008-1612 19470707

[B53] Manneras L Cajander S Holmang A Seleskovic Z Lystig T Lonn M Stener-Victorin E A new rat model exhibiting both ovarian and metabolic characteristics of polycystic ovary syndrome Endocrinology 2007 148 3781 3791 10.1210/en.2007-0168 17495003

[B54] Shaw RJ Lamia KA Vasquez D Koo SH Bardeesy N Depinho RA Montminy M Cantley LC The kinase LKB1 mediates glucose homeostasis in liver and therapeutic effects of metformin Science 2005 310 1642 1646 10.1126/science.1120781 16308421 PMC3074427

[B55] Pellatt LJ Rice S Mason HD Phosphorylation and activation of AMP-activated protein kinase (AMPK) by metformin in the human ovary requires insulin Endocrinology 2011 152 1112 1118 10.1210/en.2009-1429 21209024

